# “I don’t have options but to persevere.” Experiences and practices of care for HIV and diabetes in rural Tanzania: a qualitative study of patients and family caregivers

**DOI:** 10.1186/s12939-016-0345-5

**Published:** 2016-04-02

**Authors:** Mary N. Mwangome, Eveline Geubbels, Paul Klatser, Marjolein Dieleman

**Affiliations:** Ifakara Health Institute, Dar es Salaam, Tanzania; Royal Tropical Institute, Amsterdam, The Netherlands; Free University Amsterdam, Amsterdam, The Netherlands

**Keywords:** Chronic care, Diabetes, HIV, Access, Community care, Sub Saharan Africa, Tanzania

## Abstract

**Background:**

The high prevalence of chronic diseases in Tanzania is putting a strain on the already stretched health care services, patients and their families. This study sought to find out how health care for diabetes and HIV is perceived, practiced and experienced by patients and family caregivers, to inform strategies to improve continuity of care.

**Methods:**

Thirty two in-depth interviews were conducted among 19 patients (10 HIV, 9 diabetes) and 13 family caregivers (6 HIV, 7 diabetes). Diabetes patients and caregivers were accessed through one referral facility. HIV patients and caregivers were accessed through HIV clinics at the district hospital, one health centre and one dispensary respectively. The innovative care for chronic conditions framework informed the study design. Data was analysed with the help of Nvivo 10.

**Results:**

Three major themes emerged; preparedness and practices in care, health care at health facilities and community support in care for HIV and diabetes. In preparedness and practices, HIV patients and caregivers knew more about aspects of HIV than did diabetes patients and caregivers on diabetes aspects. Continued education on care for the conditions was better structured for HIV than diabetes. On care at facilities, HIV and diabetes patients reported that they appreciated familiarity with providers, warm reception, gentle correction of mistakes and privacy during care. HIV services were free of charge at all levels. Costs involved in seeking services resulted in some diabetes patients to not keep appointments. There was limited community support for care of diabetes patients. Community support for HIV care was through community health workers, patient groups, and village leaders.

**Conclusion:**

Diabetes and HIV have socio-cultural and economic implications for patients and their families. The HIV programme is successfully using decentralization of health services, task shifting and CHWs to address these implications. For diabetes and NCDs, decentralization and task shifting are also important and, strengthening of community involvement is warranted for continuity of care and patient centeredness in care. While considering differences between HIV and diabetes, we have shown that Tanzania’s rich experiences in community involvement in health can be leveraged for care and treatment of diabetes and other NCDs.

## Background

Similar to other Sub Sahara African (sSA) countries, Tanzania has a high burden of HIV and the prevalence of non- communicable diseases (NCDs) is rising. Thirty one per cent and 11.4 % of all deaths in the country in 2014 were attributed to NCDs [1] and HIV [2] respectively. Diabetes, one of the four major NCDs (the other three being cardiovascular diseases, cancers and chronic obstructive airway diseases [3]), was estimated to have a national prevalence of 7.95 % among persons aged 20 to 79 years in 2013 [4]. This condition was responsible for 2 % of all deaths in Tanzania in 2014 [1]. The prevalence of HIV in the general population was estimated at 5.1 % in 2012, a marked decline from 7 % in 2004 [5].

The HIV/AIDS programme in Tanzania is over 20 years old. It has a history of substantial investment that has accomplished the reduction of prevalence and deaths attributable to HIV. Through the programme, HIV prevention interventions including the promotion of condom use and the prevention of vertical transmission among other strategies, have been promoted and succeeded in reducing the rate of new infection [6, 7]. For HIV care and treatment, clinics have been rolled out to lower level facilities like health centers and dispensaries, various cadres of staff have been trained in HIV care, task shifting of clinical duties to non-clinician staff has been implemented, home based care services have been scaled up and HIV information and supplies management sub-systems are operational [7]. The availability of HIV treatment has transformed treated HIV into a chronic disease with largely similar health care needs as chronic NCDs like diabetes. Community partners like peer educators, expert patients, and home based care providers have contributed immensely in prevention as well as care and treatment efforts in Tanzania [8–11].

Government efforts to deal with diabetes are integrated within NCD national efforts although privately resourced diabetes interest groups, like Tanzania diabetes association, are contributing to advocacy and community awareness efforts [12]. The government efforts include the establishment of the NCD unit at the ministry of Health and the inclusion of improvement of both facility-based and home-based care for those afflicted by NCDs as an objective in the health sector strategic plan [13]. The government has also developed an action plan for the prevention and control of NCDs in Tanzania and included information, education and communication as strategies for NCD prevention and control in the primary health services development plan 2007–2017.

The burden of HIV and diabetes, among other chronic diseases places a heavy load on the already strained health system in the country. In a northwestern regional hospital in Tanzania, a study of 11,045 consecutive adult medical admissions between 2009–2011 found that 48 % of admissions were due to NCDs (26 % of the 48 % were due to diabetes) and 21 % due to HIV, and that almost three quarters of all hospital days were attributable to NCDs and HIV [14]. It is also known that chronic diseases place extra demands on patients, their families and their communities because most of the care for chronically ill patients takes place at community and family level [15]. These demands are increased by the fact that chronic diseases are increasingly occurring in parallel as co-morbidities or multi-morbidities even in African settings [16]. However, little is known about how care for chronically ill patients is perceived, practiced and experienced by patients and their caregivers at facility and community levels.

We searched literature to find studies from sSA on experiences and practices of patients and family caregivers regarding HIV and diabetes care both at the health facility and at the community. Following is a summary of the studies we found and their results. Studies on patient experiences with HIV care in SSA showed that, positive interactions of HIV patients with health providers led to good adherence to medication [17, 18]. Other studies revealed that perceived stigma, felt or anticipated, influenced how HIV patients sought and received care for their condition and hence influenced their adherence to medication [19–22]. Studies on experiences of diabetes patients recounted health seeking patterns of diabetes patients [23, 24]. In Ghana diabetes patients sought for cure for diabetes by moving between providers of biomedicine, ethno medicine, and faith healing, a situation that exacerbated diabetes complications [24]. Studies from Tanzania and South Africa revealed the difficulties diabetes patients go through to access diabetes health services [23, 25]. On family involvement, studies show that facilitated family level conversations on care had positive effect on HIV infected adolescents’ adherence to medication and spiritual beliefs [26, 27]. In other studies, support of family caregivers of HIV patients by health providers empowered the caregivers to care for their patients better and enhanced continuity of care [28, 29].

Regarding chronic disease care at the community (non-family persons or organizations), we found a review of studies on community health workers (CHW) (which included peer educators, lay counselors, home based care providers) in low and middle income countries (LMICs) which found that CHWs enhanced the reach, uptake and quality of HIV services but also that their roles were not clearly defined [30]. For diabetes care, studies involving community health service providers (none from Tanzania) showed conflicting findings that may be due to differences in type of community providers or in the services provided. For instance, use of peer educators to support diabetes patients’ self-care through regular meetings in Cameroon and Uganda improved blood sugar and blood pressure control [31, 32]. By contrast, a South African study found that diabetes patients cared for at the clinic had better blood sugar control compared to those in a CHW outreach programme that supplied drugs to patients at home with home based patient monitoring by CHWs [33].

Information on how patients, and family caregivers practice and experience care for chronic diseases is necessary especially for rural areas where majority of the Tanzania population resides, HIV prevalence is high [34] and the burden of diabetes and other NCDs is substantial [35]. However, compared to urban areas, health care services are not readily available in the rural areas where the health worker crisis is also worse than in urban areas [36]. Gaining insight into patients’ and family caregivers’ needs and how they cope with these needs in rural, resource constrained circumstances will inform policy makers and health planers on priority actions to improve care for chronic diseases in such settings.

**Fig. 1:**
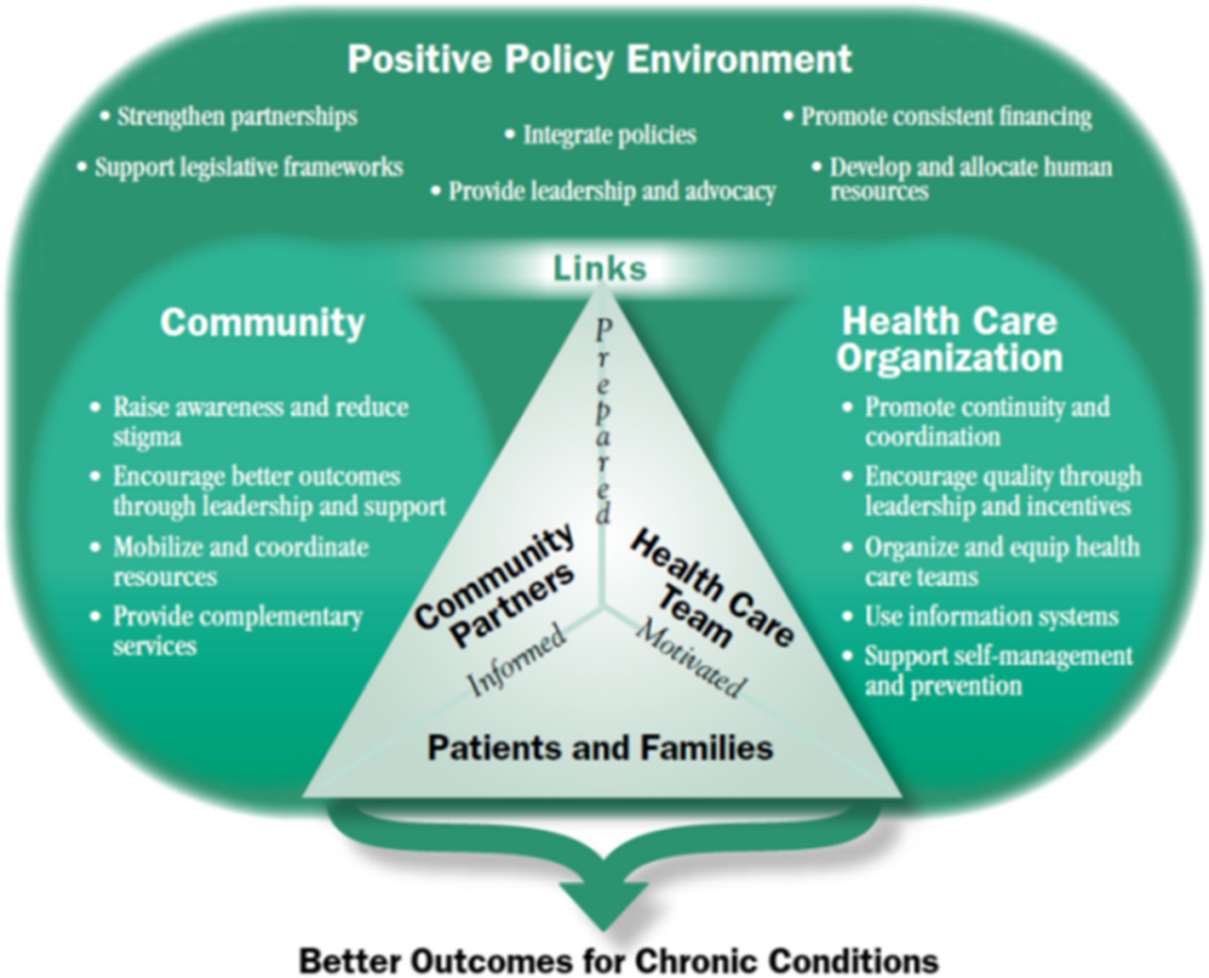
The Innovative Care for Chronic Condition framework. Source of Fig. 1: Sheri Pruit et al. 2002 [34] (open access)

The Innovative Care for Chronic Conditions (ICCC) framework [37], illustrated in Fig. 1, advocates a patient-centred approach to chronic health care with a patient interaction level consisting of three groups of players: patient with their families, community partners, and the healthcare team that are linked and well informed about the disease and its care. Together they should be prepared to manage the disease and its crises and motivated to support the patient to care for their disease. The ICCC has been used in South Africa to design strategies for dealing with NCDs [38]. Although by 2012 it had not been implemented operationally in SSA [39], we found it useful in structuring the development of this study.

Guided by the ICCC, this study seeks to find out how care for diabetes and HIV, both in the community and at the health facilities, is practiced, experienced and perceived by patients and family caregivers. The findings will be useful in informing future strategies to improve the relationships and continuity between health care for chronic diseases at health facilities and in the community in rural settings of Tanzania. We used HIV and diabetes as examples of chronic diseases because they both contribute significantly to workload in health facilities in Tanzania [14] and also because experiences from the long standing HIV programme could inform the design of care and treatment interventions for diabetes and other emerging chronic diseases. In this study, diabetes refers to type 2 diabetes.

## Methods

### Study setting

The study was conducted in September and October of 2014, in a district in Tanzania with a population of 265,203 persons, 87 % of whom reside in rural villages. In terms of public health services, it has one district hospital, three health centres and 33 dispensaries. There is only one referral hospital serving this district which is located in the neighbouring district. Regarding private health services, there are three dispensaries and one hospital, all owned by a faith based organization (FBO). The FBO-owned hospital in the study district was reported to have started offering diabetes services in 2012. The study was conducted in four facilities: the referral hospital, the district hospital, one health centre and one dispensary. Besides the referral hospital which is owned by a FBO, the other three selected facilities were public facilities.

We selected three HIV clinics from different levels in the health system (district, health centre and dispensary) to capture any differences in patients’ and family caregivers’ perceptions and experiences on care for HIV. In this study family caregivers refer to relatives or family members of a chronic disease patient who is perceived by respondents to participate in the care of the patient. The selected HIV clinics offered similar HIV care services of counselling, treatment initiation and monitoring and management of opportunistic infections. For more advanced laboratory or radiological tests or symptoms of complications that need to be managed at more specialized centres, providers at lower level HIV clinics referred patients to HIV clinics at higher level facilities. The referral hospital’s HIV clinic was not included because it was not offering typical public service as it was differently resourced (funded by research organization) and operated at clinical research standards.

We selected the referral hospital’s diabetes clinic because it was the only diabetes clinic that offered care systematically, had been established more than 8 years prior, was more typically resourced with FBO funds supplemented by Government funding, and had an information system that could facilitate identification and tracking of patients and family caregivers. The referral facility diabetes clinic had over 500 registered diabetes patients, mostly from the study district and that of the referral hospital. In both districts health centre and dispensary level facilities, public and private, did not systematically offer diabetes care services.

### Study design and sampling

We used a qualitative research design whereby in-depth interviews (IDIs) were conducted to elicit information on personal experiences and perceptions on health care for HIV and diabetes respectively. Inclusion criteria for study patients were: adults aged 18 years or more, who had been diagnosed with diabetes or HIV for two or more years at the time of the study and had been registered for care at the selected HIV or diabetes clinics respectively. For the family caregivers the inclusion criteria were; they had to be relatives of HIV or diabetes patient respectively (did not have to be patient-caregiver dyads), aged 18 years or more and the patients they cared for should have been diagnosed with the chronic condition at least two or more years earlier. We purposively selected participants using the above criteria in consultation with clinic nurses who used health records that also recorded the patients’ next of kin. Patients were approached by the clinic nurses and asked if they would like to discuss with researchers about participation in a research activity. This was done after patients had received the services they needed from the nurses. Those who accepted were then given information about the study by the researchers and asked to provide written consent if they chose to participate. Caregivers for both HIV and diabetes patients were identified by clinic nurses and selected according to the inclusion criteria. Some caregivers, who had escorted their patients to the clinic, were also approached by clinic nurses and after confirming eligibility, were asked if they would discuss participation in the study with researchers. Other caregivers were identified through patients who attended clinic without an escort. These patients were asked by clinic nurses if researchers could contact their caregivers after nurses ascertained caregivers’ eligibility. For patients who agreed, their caregivers were contacted with the patient’s help and invited to come to the clinic for interviews. For three diabetes caregivers however, interviews were conducted at their homes. The use of clinic nurses to approach potential respondents was preferred to maintain confidentiality of the patients’ condition. Participant recruitment was stopped after saturation was achieved.

### Data collection and analysis

The first author and two trained research assistants with a social science background conducted 32 interviews; 19 with patients (10 of HIV and 9 of diabetes patients) and 13 with caregivers (6 caregivers of HIV and 7 of diabetes patients) all of whom were relatives of patients. The family caregivers will henceforth be referred to as HIV and diabetes caregivers respectively. We used two topic guides for data collection from patients and caregivers respectively, which explored what they knew about the disease, how they learned to care for the diseases and for caregivers specifically, what motivated them to continue caring for the diseases and what they did to care for the patients. We also asked them for their experiences with and perceptions of the community and health providers at the health facilities regarding care for HIV and diabetes. We adapted the guides following analysis of pilot interviews. The first author checked transcripts for accuracy, anonymised and imported them into Nvivo 10 (QSR International Pty Ltd, Australia). Two authors (MM and MD) developed codes based on ICCC framework’s description of the patient interaction level of chronic care. The transcripts were analysed in their original language (Kiswahili). After coding the transcripts, we categorized the codes into the following themes: (i) preparedness for and practices on care for the conditions (ii) perceptions on and experiences with care at health facilities, (iii) perceptions on and experiences with community involvement in patient care.

### Ethical considerations

The institutional review board and the National Institute for Medical Research’s ethical committee (NIMR/HQ/R8.a/vol IX/1459 (10/1/2013) approved this study. Each participating individual provided written consent after being informed about the research. Participants who could not write provided consent by thumb printing on the consent form after an independent witness of their choice explained to them what the research was about. The witness also signed and dated the same consent form.

## Results

### Characteristics of respondents

We interviewed 32 respondents whose characteristics are presented in Table 1. Diabetes patients were on average older than HIV patients and the majority of respondents, patients and caregivers (26/32) had completed primary education or higher level.

**Table 1:** Social and demographic characteristics of study participants

Characteristic of participants		HIV patients	HIV caregivers	Diabetes patients	Diabetes caregivers	Total
		*N* = 10	*N* = 6	*N* = 9	*N* = 7	*N* = 32
Age (years)	Median	48.5	49.5	61	35	49
	Range	36 - 62	21-63	40-79	18-59	18-79
Gender	Female	5	3	5	4	17
	Male	5	3	4	3	15
Time^a^	Median	4.5	3.5	3	3	3.25
	Range	2-10	2-5	2-16	2-20	2-20
Education	Illiterate	0	1	1	1	3
	Some primary	0	0	2	1	3
	Primary completed or higher	10	5	6	6	26

^a^Patients = time since diagnosis. Caregivers = time since starting care of the patient

### Preparedness for and practices of care for HIV and diabetes

This theme encompasses the findings regarding the preparedness of both patients and family caregivers to care for HIV and diabetes respectively. The preparedness is described in terms of their knowledge and motivation, the sources of this knowledge and motivation and their practices related to care of HIV and diabetes respectively.

#### Knowledge and sources of motivation to care for HIV and diabetes

HIV patients and caregivers had knowledge of more aspects of HIV than did diabetes patients and caregivers about aspects of diabetes. The majority of HIV patients (6/10) and half of caregivers from the three levels of facilities (3/6) knew what the Swahili acronym for HIV stands for, and all respondents knew how HIV is contracted and how to prevent persons from contracting HIV.

In contrast, the majority of diabetes patients and caregivers of diabetes patients did not know how one developed diabetes and hence could not tell how to prevent it. However, most patients (7/9) and caregivers (4/7) mentioned that diabetes was increased sugar in the body. Only one diabetes patient and one diabetes caregiver mentioned that the pancreas had a problem and only the patient mentioned insulin was not working well.

*It is the pancreas that cannot make insulin is the problem of diabetes so a lot of sugar gets into the body. Insulin its work is to “balance” the sugar getting into the body but since the pancreas is dead it is not making insulin and the sugar gets into the body. (Diabetes patient, Female)*

All participating HIV and diabetes patients and caregivers knew that the respective conditions were not curable, and that patients had to continue taking medication throughout their lives to feel well. However on the course of disease, some diabetes patients (2/9) and a diabetes caregiver expressed confusion about the concept of low blood sugar in diabetes patients and why it would make them unwell, despite the disease being the result of high blood sugar.

*“You suffer a lot because you don’t [have appetite to] eat and then as a diabetes patient if you do not eat the sugar [in the body] goes up.”(Diabetes patient, Male)*

Regarding motivation, all HIV and diabetes patients reported the need to stay alive and healthy as the main motivation to continue following the instructions given for caring for their conditions. Some HIV (2/10) and diabetes (2/9) patients were motivated by the feeling of responsibility towards their dependants. Another source of motivation for most HIV (8/10) and diabetes (8/9) patients was family members who provided financial support and day to day care. For most HIV (7/10) and some diabetes (4/9) patients, health providers were motivators of patients through verbal encouragement and provision of information on care.

HIV caregivers (5/6) and all diabetes caregivers said they were motivated by their sense of social responsibility to care for a loved one. Seeing the patients they cared for do well encouraged most caregivers (10/13) to continue caring for their patients.

Both HIV and diabetes caregivers reported a range of effects of caregiving. Most commonly reported was economic hardship occasioned by patients care needs like money for transport to hospital or for medication or for special foods in case of diabetes. Other reported effects include loss of business customers due to delayed delivery of goods, having to bear with patient’s bad moods, psychological unrest that affected school work, and being accused as the cause of disease to the patient by other relatives.

*“At school I become very worried and even sometimes during prep [evening study time]I spend the whole time just thinking why am I in this situation or is God punishing me?” (Diabetes caregiver, school girl)*

*“Before I started caring for mother I used to earn about 200 to 250 thousands TZS [USD 122.6 or 153.3] in a month but since I started caring for mother I get maybe sixty thousands because I get tired and cannot finish work on time.”(HIV caregiver, Male)*

To these effects, the majority of caregivers responded by accepting the situation as it was and were not discouraged from continuing to care for their patients. A diabetes caregiver for instance explained how she had to learn to be patient and persevering in order to deal with patients’ changing moods.

*“…if you decide to stay with him that day you find he is always quarreling, he finds everything I do to be a mistake so those are the changes in him and I don’t have options but to persevere.”(Diabetes caregiver, Female)*

#### Information on care for HIV and diabetes

Information on how to care for themselves while at home was initially provided to patients by health providers at diagnosis of HIV and diabetes, according to all participating patients. The diagnosis of diabetes for most diabetes patients happened during in-patient care when patients were seriously ill, unlike for the majority of HIV patients who were diagnosed in outpatient settings at the facilities they were currently attending for HIV care. On-going education of patients and caregivers occurred on specific days for HIV. The targeted persons were those about to start antiretroviral (ARV) medication, their treatment assistants and patients who were not progressing well on treatment.

HIV patients from the three facilities expressed appreciation for the education they received from providers given the changes they had experienced as they followed the guidance.

*“Even the education they give me for example when I weigh and they find my weight has reduced they ask me why my weight has reduced, I explain to them why I think it has reduced. When I explain to them they tell me to do this and this and that education really helps me to keep my health well.” (HIV Patient, Male)*

On the other hand, complaints about lack of on-going diabetes education were made by the majority of diabetes patients (8/9). Some diabetes patients (2/9) recounted that pamphlets used to be distributed for use at home but other diabetes patients reported never having received any pamphlet. The pamphlets, according to a patient who had received one, explained symptoms of increased sugar, what to eat and how to care for feet. Diabetes patients also explained how they learnt some skills from each other while at the clinic waiting for services.

*“When we sit at the bench there [outside clinic] when we are waiting for services, you find experienced patients and when we sit with them there we teach each other how to keep the sugar down like if you want the sugar not to rise don’t use this and this because others have been treated in other places so they give us their experience so we do what we can copy from them and we do like them.”(Diabetes patient, Female)*

The majority (5/6) of HIV caregivers reported that they learnt how to support patients to care for their conditions from health providers, including through the education sessions for treatment assistants. Others reported sources of information on HIV care included HIV related non-governmental organization (NGO) offices and radios. Some HIV caregivers (2/10) claimed to have had prior experience through taking care of other chronically ill family members which added to what they learnt from providers.

*“I: How do you know how to care for this patient?*

*R: Because I took care of others, I do not see any difference with caring for his late mother.” (HIV caregiver, Female)*

Conversely, all participating diabetes caregivers initially learnt from the patients themselves or other caregivers who were with the patient at diagnosis, how to care for the patient’s condition. Caregivers described dietary restrictions as the subject about which they learnt most. For on-going learning about care, diabetes caregivers (2/7) reported that they waited until appointment day to ask health providers questions about patient care practices because of the cost implications of making unscheduled visits to the referral hospital. Otherwise, the majority of diabetes caregivers (4/7) reported that they did not ask anyone any questions regarding care of the patients because they had understood what the patients had told them.

*“I: Who explained to you how to care for him?*

*R: When he came home from hospital he told me the instructions.*

*………*

*I: And when you have questions about caring for the disease of your patient who do you ask?*

*R: I cannot ask questions because he told me everything, I understood so I follow what he told me.”(Diabetes caregiver, Female)*

#### Patient practices when unwell

Generally, HIV patients reported more use of their clinics and less of self-care practices compared to diabetes patients when unwell. Most HIV patients (6/10) said that they sought medical care at their HIV clinic for minor ailments and others reported self-medicating first with painkillers. For patients attending the dispensary’s HIV clinic, where there was no laboratory, one patient described how she went to a private laboratory first for tests on blood and urine as she would be advised by the laboratory technician, before going to the dispensary with the results for care.

The majority of diabetes patients (7/9) indicated that they could tell by how they felt that their blood sugar was not normal. Some patients (3/9) explained that minor ailments caused their blood sugar to rise and therefore had to act on the ailments swiftly. Diabetes patients told of different ways of finding out if increased blood sugar was the cause for their symptoms. For instance, some patients went to private laboratories, or to health facilities, others self-tested for blood sugar using personal blood sugar testing machines or tasted their own urine for sweetness if they did not have money for blood tests.

*“I: How do you test?*

*R: You lick your urine, if sugar is high, it will taste like tea that has a lot of sugar, you will taste it or if your urine drops on the floor you will see stickiness or ants come to it you will think it is sugar”.(Diabetes patient, Female)*

If increased blood sugar was found to be the cause of symptoms, some patients responded by buying medication if they did not have any, a few by drinking a lot of water, and others by being more careful with their diet. Only one out of four patients who were reported to be owning personal blood sugar testing machines had engaged with diabetes clinic providers in learning how to use it and in interpreting the results.

#### Caregivers’ support in patient care

According to all participants in the study, caregivers supported patients through direct assistance with day to day activities or through enabling of the care. Direct assistance was commonly reported to be offered by female caregivers and included cooking for the patient, reminding him/her to take medication, monitoring well-being, and specific to HIV caregivers, picking refill medications on some appointment visits on behalf of the patient.

*“Other than picking medications for him at the clinic, I help by washing his clothes, I fetch water, I cook food for him and remind him to take his medication.”(HIV caregiver, Female)*

Conversely, enabling of care, commonly reported for male caregivers of HIV and diabetes patients, was done through sending money to direct caregivers or patients. However, specific to diabetes care, some caregivers obtained the special foods required for diabetes patients like sorghum and other caregivers bought personal blood sugar testing machines for the patient.

### Experiences with health care at health facilities for HIV and diabetes

This thematic section presents the findings related to experiences and perceptions of patients and family caregivers regarding their health providers and the services they receive for HIV and diabetes.

#### Perceptions on and experiences with health providers

The majority of HIV (7/10) and diabetes (5/9) patients said that providers treated them well, especially the ones they were familiar with. They expressed appreciation for the warm reception and gentle correction of mistakes in care practices from providers.

*: R: At home they care for me and even here they care for me. When I come, for all the time I am here, it is like the providers are my mothers……this gives us heart to continue with medication and live like other people.”(HIV patient, Female)*

However, two HIV patients, from health centre and dispensary respectively, reported use of harsh language on them which caused hurt and discouragement such that the patients felt that they could no longer freely express their needs to the providers.

#### Service access and quality

It emerged that HIV services including medication were offered free of charge at all levels of clinics while diabetes patients reported having to pay, at every visit, a consultation fee of 4500 TZS (USD 2.75), buy their own medication at every visit and for some, incur travel and accommodation costs to attend clinic appointments. Only one HIV patient at the health centre reported having to incur travel costs to the clinic. Long distances and costs involved to reach the diabetes clinic (travel and lodging costs) caused some patients (4/9) to miss visits frequently, making the visit only when unwell. When they skipped a visit to clinic, patients reported using the latest prescription they had to buy diabetes medicines from private drug outlets, which saved them the consultation fee and transport money.

*“…….the big challenge in attending clinic is to lack money for buying medication because every time you go medication is not below 15,000/= TZS[USD 9.2] or sometimes even 20,000/= [USD 12.26]and consider that seeing the doctor before buying the medication costs 4500/=[USD 2.75]. This is why I miss clinic sometimes and when I miss, I buy medicine even half a dose using my notebook [patient notes]. I use until I get money for going to clinic.”(Diabetes patient, Male)*

Furthermore, HIV patients at all levels stated that they could send treatment assistants to collect medication refill on their behalf whereas diabetes patients explained that they could not do this because they had to be tested for blood sugar at every visit. According to some HIV caregivers (4/6), treatment assistants made the visits on behalf of their patients when patients were engaged elsewhere, tired, or just to relieve them of the trouble.

The absence of expected necessary services that are usually available was reported by some HIV (3/10) and diabetes (6/9) patients. For instance, out-of-stock ARV made HIV patients miss their ARV doses at both the health centre and dispensary clinics. For diabetes patients, the absence of prescribed medication at the hospital pharmacy which sells them at cheaper prices or lack of required eye care services that were beyond the facility’s expertise were reported. This missing of services caused anguish to patients and their caregivers as the excerpt illustrates below:

*“Now, his [the patient’s]condition is not very good. They had given him medication which he took for a while and he felt better. But now when he went to the clinic there is no medicine and they have to write [a prescription] for him to go and buy and they [medications]are more expensive there[at drug outlets in town].........(Diabetes caregiver, Female)*

Delays in HIV services were reported by patients attending the health centre and dispensary clinics, where the HIV service providers were also the general outpatient department (OPD) service providers. These patients thought that shortage of staff contributed to these delays.

It emerged that delays were also common at the diabetes clinic. Diabetes patients thought that delays were caused by the many service counters or rooms they had to go to in one visit (reception, insurance office for the insured, laboratory, clinic rooms, and pharmacy) and the late arrival of doctors to the diabetes clinic. According to a diabetes patient, these delays put them at risk of fainting since they were required to come for routine visits having fasted for the required blood sugar test.

All HIV and diabetes patients indicated that privacy during service provision was important to them. Some diabetes patients (4/9) reported that privacy was lacking in the doctors’ room where two or three doctors shared the room; patients felt that they were unable to be open with the doctor about their problems, especially personal ones. At the health centre and dispensary HIV clinics, HIV patients complained of the risk of their confidentiality being broken as they sometimes shared the waiting bay with OPD patients and their blue HIV patient cards were distinctive from notebooks that OPD patients used. HIV patients from all three clinics, did not consider the premises at which they received services to offer sufficient confidentiality and one patient at the district hospital reported knowing of a fellow HIV patient who stopped attending clinic due to fear of confidentiality being breached.

*“This place is not appropriate, everyone who comes in can see us and others discriminate against us but now we are grateful because they are putting up a new building for us which is more private.” (HIV patient, Female)*

### Community support in care for HIV and diabetes

This theme, describes the different types of non-family community actors, who actively participate in supporting care for HIV and diabetes at community level, as perceived by HIV and diabetes patients and family caregivers.

Community partners, defined as non-family community members and organizations in the community, were involved in the patients care in a variety of ways. For HIV care, community 1`partners were reported to provide informal care services. For instance, three HIV patients from the district and dispensary clinics and one HIV caregiver from the district clinic recounted that community health workers (CHWs) visited respondents’ homes. Some CHWs were reported to be linked to the HIV clinic and others to NGOs. The reported activities of the CHWs were encouraging people to test for HIV, providing care information to encourage adherence to medication or encouraging patients to call on them in case of minor ailments for treatment. Respondents indicated that they were pleased with the CHW services.

*“There are those workers of XXXX [NGO], they come home many times to check on mother and give us lessons on how to care for her. They encourage my mother a lot. I mean when they leave, I see a difference in mother, I mean she seems more lively.” (HIV caregiver, Male)*

Other forms of care services were occasionally provided by health professionals personally known to patients or caregivers. For example a nurse friend of an HIV patient visited often and offered professional advice such as what to do when a dose was missed.

Stigma emerged as an important barrier for involvement of friends and neighbours in care for HIV patients. Whereas some patients explained that they had disclosed their HIV status to some friends and received support such as encouragement to continue caring for themselves, three HIV patients reported some experiences with stigma on themselves and on fellow HIV patients that caused them to fear disclosing their HIV status.

*“There is one lady who is a neighbour who decided to say with her mouth and tell me “I can’t talk to you infected person”. I felt very bad. She decided to tell me that when we were talking about something very different. Her son was making noise in the area and I went to ask her to make the boy stop. Then in anger she decided to tell me that.” (HIV patient, Male)*

Two HIV patients and one HIV caregiver, all attending the district hospital’s HIV clinic, explained how village leaders visited their homes to encourage them, how the leaders protected patients against stigmatizing neighbours and how they exempted HIV patients from some community responsibilities like hard-labour activities. Both facility-based and community-based patient support groups, according to some HIV patients, also offered an opportunity for patients to discuss challenges and how to overcome them and encouraged each other to continue adhering to medication and other care practices. Some HIV patients attending district and dispensary clinics (5/10) also reported that community-based patient groups supported by NGOs and the social welfare department, independently, provided assistance to facilitate income-generation for patients. Patients were asked to form groups and open a group bank account into which the funding organization deposited some money for group members to start up small businesses. These businesses aimed to generate some money for patients’ day to day use and also for repaying the loan. Patients expressed appreciation for this support.

For diabetes patients, there were no experiences reported with CHWs. Informal diabetes care services were reported by one diabetes patient and one diabetes caregiver. For example, a nurse neighbour assisted the caregiver in performing and interpreting blood sugar tests and advised accordingly.

*“If we see his condition has changed we go to the nurse because we are neighbouring here, we call her she comes and tests the patient. If she sees may be the sugar is high she advises us on what to do or should we take him to hospital?”(Diabetes caregiver, Female)*

Neighbours also supported primary caregivers with caring for diabetes patients when the caregiver had to be away, according to some diabetes caregivers (4/7). When leaving for a whole day or longer, they left neighbours to look after their patients, to monitor the well-being of the patient, and ensure that the patient took their medication and food on time. Regarding patient support groups for diabetes patients, there were varying reports on the existence of such a support group with two diabetes patients reporting of the existence of one, one diabetes patient stating that he had heard of its existence but had not attended its meetings and majority of diabetes patients saying that they had never heard of it.

## Discussion

Our study has highlighted the variations between HIV and diabetes care in terms of patients and caregivers’ knowledge of disease and its care, physical and financial access to services and involvement of community partners. Interpretation of these findings requires consideration of the socio-cultural and economic implications that these diseases have on patients and their families which may vary for different chronic diseases. The social and cultural implications arise, for instance, with diabetes affecting older people than HIV [40] as is also reflected by our study sample, with implications on the kind and amount of care the patient requires. Besides affliction by chronic NCDs, older persons also tend to be physically weaker and frail due to the ageing process which further increases their care needs [41]. The heavy responsibility of caring for such ill persons falls on the patients’ families according to the African traditions of family and community solidarity [41]. This could explain our finding that family caregivers felt socially responsible to care for their patients. Furthermore our study findings also demonstrate that the day-to-day care for chronic diseases patients was shouldered by women. Although both diseases require patients to adjust to healthier lifestyles like healthy eating, diabetes requires more intrusive and active self-management in terms of dietary adjustments. These intrusive adjustments have socio-cultural implications in terms of effects on the family dietary pattern like the type of foods to cook and cooking for the whole family versus having a separate pot for the patient. In addition, different communities have pre-existing food traditions and taboos [42] that would need to be considered when planning a dietary regime for a diabetes patient. Conversely, HIV patients are confronted with stigma which diabetes patients do not experience as our study also showed [39]. The social implications of stigma include blame, prejudice, and discrimination of the stigmatized person(s) [43]. For chronic diseases, culturally ascribed aetiologies of the diseases sometimes leads to abandoning biomedical management or using traditional medicine together with biomedical treatments with implications on health outcomes for such patients [44].

The economic implications of both HIV and diabetes are in terms of access to health services, medication and prescribed foods. The longevity of the HIV programme in Tanzania and the benefits in terms of enormous technical and financial support from international partners (currently at about 500 million USD annually) [7] has enabled HIV patients to access HIV care services, including medication, free of charge. Services for diabetes patients on the other hand, like we found in our study, remain centralized in larger health facilities, which imposes a financial cost on patients and their families to access services.

For chronic diseases, the way the services are organized and delivered matters in ensuring access to, acceptance and continued utilization of the services. One modality of organizing the delivery of services is decentralization. Decentralization of HIV care and treatment services to lower level facilities and the sharing of tasks originally undertaken by clinicians, like prescribing, to non-clinician cadres like nurses, were adopted by the HIV programme in Tanzania to alleviate the economic implications. Indeed, the decentralization and task shifting have increased the number of HIV patients accessing HIV treatment services from about 2000 in 2003 to over 660,000 in 2012 [7, 45]. For diabetes and other NCDs, given our findings that cost implications caused reduced utilization of health services, decentralization of health services to lower level health facilities and task sharing would also be justified to address the economic burden on rural patients. There are efforts towards this in Tanzania whereby through a foreign grant, some health centres in the lake region of Tanzania had diabetes clinics set up, equipment provided and staff trained for diabetes care [46, 47]. We, however, could not find records of evaluation of these efforts. Studies in Cameroon, Ethiopia and South Africa have nonetheless shown that decentralization and task shifting of diabetes and hypertension services to rural health centres and dispensaries operated by nurses is possible through training, use of simple equipment and of diagnostic and treatment protocols. The outcomes of the decentralization and task shifting in these countries include acceptable quality of services in Ethiopia, an average decrease in fasting blood sugar of 3.4 mmol/L in Cameroon over two years and blood sugar control of over 80 % diabetes patients over two years in South Africa [48–50]. The ICCC framework suggests that patients and their family should be well informed, prepared and motivated to care for the chronic disease. Our findings on the sources of information for diabetes and its care, point to weak organization of information-giving to patients and their caregivers. As studies have shown that Tanzanian health providers in rural lower level facilities have less knowledge about diabetes and its management compared to their urban counterparts and compared to knowledge on HIV [51], it is important to empower facility-based health providers with evidence based knowledge of care for diabetes for them to provide high quality diabetes services which include better support for patients and caregivers to understand and manage their disease for better patient outcomes.

Whereas decentralization of services to lower level health facilities is necessary, this approach still relies on the already strained resources at these facilities. Involvement of communities in care for these chronic diseases is therefore warranted and can also provide an opportunity to address more locally the socio-cultural and economic implications of these diseases. Involvement of the community is also advocated by the ICCC framework as part of the micro-level actors who together with health providers and patients with their families should work towards better patient outcomes. Community involvement in health in Tanzania has its history in the post-independence self-reliance agenda when village health workers (VHW, a kind of CHW) working out of community health posts were key providers of primary health care with supervision from providers at dispensaries [52]. Currently, the primary health services development programme (PHSDP) 2007–2017 [53] and the draft action plan for the prevention and control of NCD in Tanzania for 2015–2020, recommend the use of CHWs to support health promotion, NCD prevention and care activities [54]. A 2012 review of Community Health worker (CHWs) (lay person, patient or not, without formal medical/clinical training, who provides health services) programmes in Tanzania found that ongoing CHW activities were largely related to HIV, Malaria, maternal and child health interventions [55]. While we could not find publications on outcomes of CHWs’ activities in HIV care and treatment from Tanzania, evidence from other countries in SSA indicate that involvement of CHW in HIV treatment adherence counselling, delivery of HIV treatment to patient homes, detection of side effects and in referral of sick patients had positive impacts on: patients’ access to care and treatment; adherence to HIV medication; retention in care; and survival [56–58]. In addition, there were also benefits at health facility level where CHWs helped in triaging, counselling and documentation which resulted in reduced work load on professional health providers. This reduced workload was also due to reduced frequency of patient visits because of on-going CHW activities [56, 58].

Given the similarities in the need for continuity of care among chronic diseases, it is logical to believe that CHWs, if well-prepared and engaged in care and treatment for diabetes and other NCDs in rural settings, can also improve patient outcomes and health facility processes. Embedding the community health roles for diabetes and other NCDs within existing roles of CHWs in HIV care, will enable better identification and addressing of locally specific socio-cultural and economic care needs. For example, it could help identify local traditional practices that are harmful to the patients. Moreover, the expansion of CHW to include care for diabetes and other NCDs will make them better able to address care needs of various chronic diseases as more patients are presenting with chronic co-morbidity [16, 59]. In this expanded role, CHWs would undertake similar functions as for HIV care and treatment but expand them to diabetes care and treatment. These functions include knowledge and information dissemination to patients and caregivers regarding diabetes and its management, disease and treatment monitoring, self-management support in lifestyle changes and referral of patients to the health facilities. To facilitate these functions, simple, existing technologies can be employed for sharing information with patients, for simpler testing and for support and supervision of CHWs by facility-based health providers [60, 61]. These technologies would also ease the interactions and exchange of information between the ICCC micro-level actors which can contribute to better patient outcomes.

Involvement of patients’ families and other caregivers in patient care is important for continuity of care for chronic diseases as suggested by the ICCC framework. The HIV home based care (HBC) programme delegates duties of caring for caregivers to the community based HBC providers (a kind of CHW) who are expected to visit patients’ homes, educate caregivers on how to care for patients and provide counselling to help caregivers deal with caregiving burden [62, 63]. For diabetes and other NCDs, CHWs may undertake the education of caregivers on management of diabetes and its crises and continued support of family caregivers to deal with the effects of caregiving. This may also reduce the involvement of informal caregiving which may be risky to patients, as closer family will be skilled and have more accountable CHWs within the community from who they can seek assistance when needed.

This study has presented a case for dealing with the socio-cultural and economic implications of chronic conditions like HIV and diabetes in rural settings through changes at the health system, community and family levels. Current policies provide an enabling environment by recommending the use of CHWs in diabetes and other NCDs-related prevention and control activities. Our findings feed into the strategies to operationalize these policies.

### Study limitations

Extrapolating the findings of this study to other rural settings should be done cautiously because the HIV and diabetes clinics selected may not be representative of other rural settings. We selected patients and family caregivers through health facilities which could underrepresent those who do not regularly use health facility services. Some information bias may have been introduced in our study by using only in-depth interview accounts of respondents without triangulation with perspectives of health providers or other methods like observation of patients at facility or reviewing records of attendance. The fact that we did not interview health providers means that we could not effectively explore the roles of health providers who are the third pillar in the ICCC-proposed triad of patients with their families, the health care team and the community. Although we used clinic nurses to select and introduce potential respondents to researchers in order to protect patients ‘confidentiality, this strategy may have led some respondents to either feel coerced or to find it difficult to criticize the services they received in their responses.

## Conclusion

The HIV programme in Tanzania is over two decades old with a lot of experience in expansion of care and treatment services to lower level health facilities and the community with successes in reducing HIV incidence and prevalence. While considering differences between HIV and diabetes, decentralization of diabetes health services to lower level health facilities and task shifting are also important. However for patient centred care and continuity of diabetes care, community involvement through expansion of roles of existing CHW cadres to include diabetes care is important and can enhance integration of services. The ICCC micro-level description can guide the organization of primary care diabetes care at facilities and in the community. However the roles of the different actors should be clearly elaborated and for each role, the actors should be empowered for effective interactions that can yield good patient outcomes.

## Abbreviations

LMICs: Lower Middle Income Countries
SSA: Sub-Saharan Africa
NCDs: Non-Communicable Diseases
HIV: Human Immunodeficiency Virus
CHWs: Community Health Workers
ICCC: Innovative Care for Chronic Conditions
IDIs: In-Depth Interviews
ARV: Anti-Retroviral
NGO: Non-Governmental Organization
TZS: Tanzanian Shillings
OPD: Out Patient Department
VHWs: Village Health Workers
